# Current scenario and challenges of clinical pharmacists to implement pharmaceutical care in DRG/DIP payment hospitals in China: a qualitative interview study

**DOI:** 10.3389/fpubh.2024.1339504

**Published:** 2024-02-20

**Authors:** Suyu Gao, Xuanxuan Wang, Yun Lu, Yunkun Liu, Qiaoli Jiang, Jiajia Feng, Weihua Kong, Likai Lin, Hong Cheng

**Affiliations:** ^1^Department of Pharmacy, Zhongnan Hospital of Wuhan University, Wuhan, China; ^2^Hospital Management Institute of Wuhan University, Zhongnan Hospital of Wuhan University, Wuhan, Hubei, China

**Keywords:** Diagnosis-Related Group payment system, Diagnosis-Intervention Packet (DIP) payment system, clinical pharmacists, China, healthcare policy, qualitative interview

## Abstract

**Purpose:**

The Diagnosis-Related Group (DRG) or Diagnosis-Intervention Packet (DIP) payment system, now introduced in China, intends to streamline healthcare billing practices. However, its implications for clinical pharmacists, pivotal stakeholders in the healthcare system, remain inadequately explored. This study sought to assess the perceptions, challenges, and roles of clinical pharmacists in China following the introduction of the DRG or DIP payment system.

**Methods:**

Qualitative interviews were conducted among a sample of clinical pharmacists. Ten semi-structured interviews were conducted, either online or face to face. Thematic analysis was employed to identify key insights and concerns related to their professional landscape under the DRG or DIP system.

**Results:**

Clinical pharmacists exhibited variable awareness levels about the DRG or DIP system. Their roles have undergone shifts, creating a balance between traditional responsibilities and new obligations dictated by the DRG or DIP system. Professional development, particularly concerning health economics and DRG-based or DIP-based patient care, was highlighted as a key need. There were calls for policy support at both healthcare and national levels and a revised, holistic performance assessment system. The demand for more resources, be it in training platforms or personnel, was a recurrent theme.

**Conclusion:**

The DRG or DIP system’s introduction in China poses both opportunities and challenges for clinical pharmacists. Addressing awareness gaps, offering robust policy support, ensuring adequate resource allocation, and recognizing the evolving role of pharmacists are crucial for harmoniously integrating the DRG or DIP system into the Chinese healthcare paradigm.

## 1 Introduction

Healthcare spending has been on the rise in most countries in recent years (1). Cost control has become a major concern in global healthcare. The two payment patterns of medical insurance expenses, DRG and DIP, have been implemented in many pilot cities in China (2). The reform of medical insurance expense payment methods for inpatients will have a significant impact on the management of medical insurance funds and the operation of medical institutions. Inpatient expenditure constitutes the largest portion of healthcare costs, therefore, the focus of cost control is increasingly shifting toward managing the expenses incurred by inpatient members.

The number of hospitals implementing DRG/DIP payment in China is increasing. Currently, more than 30 provinces and autonomous regions in China are either experimenting with or formally implementing the DRG payment methods (3). This is particularly evident in large third-class A hospitals and urban medical insurance systems.

In the process of DRG/DIP payment system reform, the participation and support degree of various personnel in medical institutions is an important factor affecting the reform of DRG/DIP payment system, and clinical medical personnel play a key role in the correct grouping of DRG/DIP (4, 5). The cost of medicine has a great correlation with the level of medical and health costs, which is an important factor affecting medical costs. Foreign research results show that pharmacists can significantly reduce patients’ medical costs by participating in DRG disease group or drug management in clinical pathways, including reduced hospital stay, average daily hospitalization cost and readmission rate (6, 7). However, domestic researches on the practical application of pharmacists in DRG are relatively few. To the best of our knowledge, there is a lack of study on the perspectives of clinical pharmacists involved in implementing DRGs/DIP payment hospitals.

In order to investigate current situation of pharmaceutical care for clinical pharmacists to implement pharmaceutical care under the DRG/DIP payment, we conducted a qualitative study to explore the development of pharmaceutical care in the environment of medical insurance payment reform.

## 2 Methods

### 2.1 Overview

We conducted in-depth interviews with clinical pharmacists to better understand their cognition of the way of medical insurance payment reform, the current status of participation and the difficulties and challenges they encountered (8). This qualitative study was approved by the Ethical Approval for Clinical/Scientific Research Project under Medical Ethics Committee, Zhongnan Hospital of Wuhan University. All clinical pharmacists provided verbal informed consent. The Consolidated Criteria for Reporting Qualitative Research (COREQ) guidelines were followed.

### 2.2 Sampling

We identified a purposive sample of at least 3 years working experience clinical pharmacists and sent out invitations asked if they would be interested in participating in an interview study between August 2022 and January 2023. Those who responded positively received an informational letter about the interview study. And asked these potential participants to provide additional information, confirm their participation, and settle a date and time for the interview. The final data were drawn from 10 individual semi-structured net-meeting or face to face interviews (They are from Beijing, Wuhan, Nanjing, Guangdong, Luotian, Shiyan and Maoming). Our convenience sample of participants was comprised of seven women and three men aged 28–50 years.

### 2.3 Interview

We developed an interview guide which included both type and order of questions, to ensure a non-threatening introduction to, and tone during, the interviews. Oral informed consent was obtained from all participants before interview. Each interview lasting 1–1.5 h. All interviews were audio-recorded with the respondent’s permission and transcribed for later analysis.

In the introduction, a standardized set of outline was used to explain the background, the overall aims, and the methods of the project. The discussion, however, was not restricted to these questions and participants were asked to talk freely about their experiences and perspectives the interviewer also answered questions that the researchers might have. The interview itself had four phases (Table 1). Of note, all of participants accepted payment.

**Table 1:** Interview phases and questions.

Phase	Interview guild
Phase I	What do you think is the DRG/DIP payment? What was your source of information?
Phase II	Do you think DRG/DIP payment have impacts on the pharmaceutical care of hospital? What pharmaceutical care are currently provided by your hospital? Please be as concrete and give as many details as possible. What were your concerns and challenges at work? Can you share your experience about this point? Do you think the pharmaceutical care currently provided is helpful to control hospital costs?
Phase III	Please share your opinions on the application of drug economy in pharmaceutical care. Was anything challenging or helpful?
Phase IV	Please give any of your opinions about the development of hospital pharmacy? Did we forget something? Do you have something to add?

DRGs, diagnosis related groups; DIP, diagnosis-intervention packet.

Upon aggregating the data, we identified two main themes: (1) Current situation of clinical pharmacists participating in medical insurance payment management, (2) Challenges faced by clinical pharmacists under DRG/DIP payment (Table 2). While numerous factors influencing the execution of clinical pharmaceutical care have persistently existed, certain factors have been amplified following the implementation of DRG/DIP payment reforms in healthcare institutions.

**Table 2:** Overview of the subcategories and categories describing the current situation and challenges of pharmaceutical services provided by clinical pharmacists in DRG/DIP hospitals.

Themes	Sub-themes
Current status of participation in medical insurance	Awareness of the DRGs/DIP payments The role of participating in medical insurance Role recognition Medical insurance pays for pharmaceutical services
Challenges faced by clinical pharmacists under DRG/DIP payment	Knowledge and skills The enthusiasm of participating in clinical pharmaceutical care Resources for clinical pharmacy services Resources for clinical pharmacy services

### 2.4 Data analysis

Each interview was digitally recorded and transcribed verbatim for analysis. Analysis involved three steps: immersion (reading and rereading transcripts to become immersed in the data), coding in Nvivo 12 plus (assigning descriptive codes to appropriate segments of text), and thematic analysis (identification of salient themes based on content and interpretative analysis). We did not calculate any measurements of inter rater agreement as we are not aiming for a quantitative analysis, but wanted to distinguish qualitatively the different opinion from each other.

## 3 Results

We reached out to 14 clinical pharmacist who met inclusion criteria and were eligible for interview participation, resulting in the completion of 10 interviews (71.4%). These interviews included 2 men (20%) and 8 women (80%), with 6 (60%) participants being younger than 35 years old. Regarding professional titles, 3 held junior positions (30%), while 7 had intermediate titles or above (70%). All participants had over 3 years of work experience. Among the clinical pharmacists, nine were affiliated with tertiary hospitals, while one worked in a secondary (county) hospital. Self-reported data collection was utilized from all demographic information. The demographic characteristics of the participating clinical pharmacists are presented in Table 3.

**Table 3:** Demographic characteristics of the clinical pharmacists (*n* = 10).

Characteristics	*n* (%) (*n* = 10)
Age	
25 to 30	2 (20%)
30 to 35	4 (40%)
Younger than 35	4 (40%)
Years of work experience	
5 to 10 years	7 (70%)
10 to 15 years	2 (20%)
More than 15 years	1 (10%)
Sex	
Female	8 (80%)
professional title	
associate professor of pharmacy	2 (20%)
pharmacist-in-charge	5 (50%)
Junior pharmacist	3 (30%)
Medical insurance payment	
DRGs payment hospitals	8 (80%)
DIP payment hospital	3 (30%)
Type of hospital	
Specialized hospitals	3 (30%)
general hospital	7 (70%)

### 3.1 Current situation of clinical pharmacists participating in medical insurance payment management

#### 3.1.1 Awareness of the DRG/DIP payments

In this study, approximately half of the surveyed clinical pharmacists exhibited insufficient familiarity with DRG/DIP payment principles. For certain pharmacists, the concept of DRG/DIP payments was entirely unfamiliar prior to the interview. This knowledge gap was notably more pronounced among pharmacists affiliated with secondary (county) hospitals and those who began working during a later phase of implementation. Reflecting this, one clinical pharmacists stated, “*I do not quite understand the meaning of DRGs, while I understand it roughly after listening to the explanation. But I still do not understand the detail*s.”(Participant 3).

The other half of clinical pharmacists, working in hospitals that imlemented DRG payment earlier, demonstrated familiarity with its details.

*“Because DRG payment are comprehensively implemented in Beijing in this year, our hospital has also implemented it. Before piloting DRG payment, we often carry out pharmaceutical care training, which involved to DRG. Our pharmaceutical care may also want to take DRG payment as an opportunity.”* (Participant 6)

During the interviews, half of the pharmacists were able to accurately described the commencement of DRG payment implementation at their hospitals., while the remaining respondents were uncertain about the exact timing. Knowledge or training regarding DRG/DIP was primarily acquired through pre-implementation hospital training. This highlights a deficiency in awareness regarding medical insurance payment matters. Clinical pharmacists within medical institutions thus demonstrate a lack of understanding concerning DRG payment and the adequacy of their training in this regard.

*“I mainly learned about it through our pharmaceutical conference, which invited experts from the Medical Insurance Bureau or professors in DRG to conduct training and report.”* (Participant 6)

#### 3.1.2 The role of clinical pharmacists in medical insurance payment management

Clinical pharmacists hold varying perspectives regarding the efficacy of current pharmaceutical care in controlling hospital medical insurance costs.

Some pharmacists believe that various measures, including pre-prescription evaluation, prescription consultation, medical insurance reimbursement criteria, clinical pathways, and pharmaceutical care interventions, contribute to effective control of indicators such as drug proportion.

*“The effectiveness of medical insurance cost control is still uncertain, because it is a long-term process, such as clinical pathway development, which needs to be reviewed, and the results are still uncertain in the slow progress, but we are still actively doing these things. I feel that there are some results in controlling costs, for example, the proportion of adjuvant drugs is obviously decreasing.”* (Participant 4)

However, contrasting viewpoints exist. Some pharmacists are less certain about the effectiveness of their involvement, emphasizing the extended duration required for participation to exhibit results.

#### 3.1.3 Role recognition

Following the implementation of DRG/DIP payment mode reform in hospitals, a prevailing sentiment among most pharmacists is that it has indeed impacted the nature of pharmaceutical care, with the most pronounced change being the alteration of clinical pharmacists’ roles. Some pharmacists have expressed uncertainty surrounding their job responsibilities. On the one hand, there’s a need to support clinical pharmacy-related services; on the other hand, due to the requesities of medical insurance payment, their roles tends to lean toward regulation. Thus, harmonizing the relationship between these two facets is essential.

*The role of the pharmacy department has two roles, one is to assist doctors to carry out rational drug treatment, and the other is to act as a regulator. So on the one hand, it's important for pharmacists to think like doctors. The second aspect is to consider the problem from the perspective of regulators. So it's sometimes a little bit of a conflict, and it's basically sort of reconciliating these two relationships*. (Participant 4)

Additionally, suggestions from some pharmacists revolved around refining the requirements for pharmacists, either as professional and technical experts or as management personnel. Others emphasize the significance of active involvement in clinical diagnosis and treatment as core tasks for clinical pharmacists.

*“I also prefer to work as a hospital medical team. Management related work occupies a lot of time, which cannot be better to clinical service work.”* (Participant 9)

#### 3.1.4 Medical insurance covers pharmaceutical care

Concerning pharmaceutical care covered by medical insurance, numerous hospitals have introduced pharmaceutical care services within their outpatient departments, although some components are still in the preparation stages. The primary focus of pharmaceutical care involves providing medication consultation to patients. However, as indicated during to the interviews, pharmaceutical clinic visits are generally limited in number, and a majority of basic drug consultations are offered free of charge. This trends stems from a lack of awareness about clinical pharmacy among both physicians and patients, thus impeding the seamless implementation of these services. The primary means of attracting patients to these services often relies on recommendations from familiar doctor or coordination with outpatient guidance.

*"We have also tried to use this WeChat circle of friends, and also published on the hospital website, related publicity, weekly meetings, to attract patients, but the effect is not very good, I think one of the main ways for this patient to find pharmacists is through doctors.”* (Participant 2)

### 3.2 Challenges for clinical pharmacists under DRG/DIP payment

#### 3.2.1 Professional knowledge and skills

The majority of clinical pharmacists interviewed acknowledged the necessity of enhancing their professional capabilities within this new payment system. The consensus among the interviewees was that the knowledge they have acquired often remains theoretical and fails to convincingly communicate with doctors from a clinical application perspective. Several interviewees even recounted instances of feeling embarrassed and helpless when confronted with a patient’s consultations, unable to provide effective solutions to their concerns.

*“We still need to learn more about some clinical knowledge, because now when patients come to inquire, they do not simply ask you about this drug problem, which is mixed with some medical knowledge, so we can only improve your own knowledge reserve, knowledge and skills, you can really solve the problems of patients. Otherwise, you say that other patients simply come to you and ask how to take this medicine, and you say that I will help you to read the instructions. I think our knowledge level is too superficial and has no connotation.”* (Participant 5)

The implementation of the DRG/DIP payment reform imposes novel requisites on the scope of work undertaken by clinical pharmacists. This includes involvement in clinical pathway development, necessitating that clinical pharmacists grasp the conceptual essence of DRP/DIG and understand comprehend the fundamental principles of disease measurement. *“A lot of clinical pathways need to be reviewed and made, so it feels like a lot of work. Then, we sometimes have a headache about the choice of drugs.”* (Participant 7).

#### 3.2.2 The enthusiasm of engaging in clinical pharmaceutical care: motivations and challenges

When considering the motivations behind pharmacists’s commitment to pharmaceutical care on a daily basis, most pharmacists emphasize that gaining recognition from both patients and clinicians serves as a significant driving force in the process of actively resolving clinical issures. Participation in clinical diagnosis and treatment processes provides a means for them to recognize their own value. Additionally, continuous learning and the accumulation of experience during their work contribute to the enhancement of their problem-solving ability. Moreover, certain clinical pharmacists are driven by a genuine passion and reverence for their profession.

*“I also mentioned that my work has been recognized by the leadership, and I also gave great encouragement to my enthusiasm for work.”* (Participant 6)

However, some pharmacists also mentioned that not all of them are primarily incentivized by performance-related factors. One clinical pharmacist expressed his struggle with his work responsibilities, stating:

*Due to the lack of clinical pharmacists, I also need to distribute drugs in the pharmacy, and the work content is monotonous and does not involve professional knowledge, so I feel that I am not motivated to work*. (Participant 3)

Assessing the performance clinical pharmacists based on the outcomes of scientific research is also among the factors that impact clinical pharmaceutical care. However, when scientific research is mentioned, negative feedback is often received. Most pharmacists expressed concerns regarding their involvement in scientific research. They noted that publications and fundings are prequisities for advancing in their professional roles, leading to considerable pressure. The predominant obstacle they identify is that clinical duties con sum the majority of their time, leaving them with insufficient energy for scientific research pursuits. Some pharmacists also admit to having lost touch scientific research due to prolonged disconnection, resulting in a sense of confusion and disorientation. Furthermore, they find themselves grappling with the dilemma of choosing between clinical research and basic research.

*Perhaps I haven't been exposed to literature and research for too long, so I don't have much inspiration*. (Participant 1)

Some pharmacists expressed a desire to integrate clinical pharmaceutical care with scientific research, aiming to enhance collaboration with doctors. Simultaneously, they emphasized the need for the pharmacy field to incorporate the development of multiple disciplines and foster cooperation across various aspects to achieve more comprehensive development.

*Some feedback from clinicians would be better, so I think one is that the contradiction between clinical research and basic research is also a problem, so I hope that clinical pharmacists can still do some clinical research, so as to have a lot of good cooperation with doctors*. (Participant 4)

#### 3.2.3 Resources for clinical pharmacy care

The core assessment indicators of DRG payment require the active involvement and guidance of clinical pharmacists. With the goal of reducing treatment expenses, DRG payment incentivizes doctors to proactively select or even discontinue the usage of certain innovative drugs. Instead, they are inclined to opt for medications that is most cost-effective from a health economics point of view. Nevertheless, respondents indicated their limited knowledge in this area and expressed a desire for more platforms to help them improve.

*“We clinical pharmacists to very systematically master the research methodology in this field may be a little difficult, is a little understanding.”* (Participant 10)

Respondents noted that understaffed pharmacists and insufficient clinical training limited the reach of pharmaceutical care. In addition, respondents frequently highlighted the need for substantial resources to help them effectively implement and advance current clinical services. These resources encompass guidelines for implemention of clinical pharmaceutical care, as well as formal evaluations of clinical pharmacists.

*“The update of knowledge is relatively fast, and I think we are not very timely for the acquisition of new guidelines or some new treatment methods. Maybe I think in the past, like in some relatively large hospitals, there may be frequent opportunities for such communication.”* (Participant 1)

#### 3.2.4 Importance of governmental and hospital policy support

Governmental and hospital policy support was seen as crucial to bolster the implementation of clinical pharmaceutical care. Many clinical pharmacists believe that it is difficult for physicians to change habits of medication without the governmental and hospital support. After the implementation of the DRG/DIP payment reform in hospitals, due to policy requirements and cost control, the pharmacy department has gained legitimate authority in managing the rational use of medications, which has increased compliance in clinical departments.

*"As the importance of clinical pharmacy increases, policies for pharmaceutical care need to be further improved."* (Participant 6)

Certain pharmacists have raised an important point regarding their engagement in rational drug use management. They express concerns that the current performance management system might have implications for their income, and subsequently, this could influence their enthusiasm in delivering pharmaceutical service. The performance evaluation of clinical pharmacists is more based on their scientific research results than the effect of pharmaceutical services, which to some extent weakens the enthusiasm of pharmacists to provide pharmaceutical services to patients

*"If a clinical pharmacist takes too many intervention measures, it can affect their own and the doctors' income. ""We are suffering from the lack of quantification, for example, if I do scientific research, publish an article, I may get a very considerable reward, then why should I go to the ward round?”* (Participant 5)

## 4 Discussion

To our best knowledge, this is the first qualitative exploration to investigate the current status and challenges of implementing pharmaceutical care by clinical pharmacists in hospitals operating under the DRG/DIP payment system in China. Participants provided invaluable from their first-hand experiences, illuminating the current status, barriers encountered, and their aspirational call for policy support. Compared with previous studies, this study paid more attention to the influence and challenge of clinical pharmaceutical care under DRG/DIP payment reform.

Interview data indicates that even in the DRG pilot cities, there were still many clinical pharmacists who did not know about it. Many interviewees stated that they were not directly involved in healthcare payment-related work, but in reality, the majority of pharmacists’ job responsibilities are indeed related to it. Through interviews, clinical pharmacists in tertiary hospitals have a clearer understanding of their roles and requirements than clinical pharmacists in secondary hospitals. They are also more motivated to work, which is consistent with the conclusions of previous studies (9). This is mainly related to the different levels of clinical pharmaceutical care system construction in hospitals of different grades (10). In the context of medical insurance payment, clinical pharmacists do experience role ambiguity and role conflict, which is similar to the previous research findings on the role cognition of Chinese clinical pharmacists (11, 12). Due to factors such as differences in economic development and healthcare systems, there are significant differences between clinical pharmacists in China and community pharmacists in Western countries (13, 14). Many foreign countries have long attached importance to the role of clinical pharmacists in medical insurance, so these countries have formulated corresponding laws and regulations (15–18). Therefore, the role of clinical pharmacists in medical insurance management should be highly valued, and the job responsibilities should be clearly defined. And it is necessary to increase policy advocacy and interpretation to promote DRG/DIP reform. In China situation, the management mechanism of DRG/DIP provides the source power for rational drug use. Clinical pharmacists need clarify their role positioning and play the role of participants and enablers in the payment reform.

In the implementation of DRGs, clinical pathways play a crucial role in standardizing patient care procedures, ensuring high-quality and cost-effective medical services (19, 20). The rationality of pharmacotherapy within these pathways is critical as it directly impacts treatment efficacy and patient safety, contributing to cost control (21). Pharmacists in the implementation process should provide optimal drug treatment plans based on clinical guidelines and latest research, overseeing drug interactions and side effects to ensure patient safety, and participating in drug selection with cost–benefit considerations (22, 23). However, our study found that pharmacists interviewed were only partially involved in clinical pathways, like reviewing drug treatment plans, and faced challenges due to insufficient pharmaceutical knowledge. This indicates a need for enhanced pharmacist participation and improved capabilities in selecting effective, safe, and economical drug therapies.

As the DRG/DIP healthcare insurance payment reform progresses, there is an increasing focus on applying pharmacoeconomic methods or conducting comprehensive clinical evaluations of drugs as references for adjusting hospital prescription lists, thereby reducing medical costs (24). In recent years, multi-criteria decision analysis has been increasingly used in these comprehensive clinical evaluation processes (25, 26). The mastery and application of these methods by pharmacists are crucial for enhancing the quality of pharmacy management. However, pharmacists in this study barely mentioned mastering these techniques, possibly due to their educational backgrounds in hospital settings, indicating a need for further relevant learning or training.

With the extensive promotion of the DIP/DRG payment, it is necessary for clinical pharmacists to be familiar with the principles of medical insurance policy, help medical staff choose better treatment plans, regulate the selection of drugs in the clinical pathway so on. Although professional competence is the core competence of all clinical pharmacists, it is known from interviews that most pharmacists often lack confidence in their clinical skills. They have indicated a willingness to implement pharmaceutical care, but are restricted by limited knowledge and skills, as well as by underdeveloped pharmacy education. There some reported that lack of additional resources and training have hindered the implementation of clinical pharmaceutical care (27). Currently, MoH has established a 1-year clinical pharmacy training program for practicing pharmacists in China, but in the DRG/DIP payment hospital, most pharmacists need to acquire policy information and expertise through their own learning or through various training programs (28). These reflect the need for improvement in training models. However, the more urgent need for pharmacy graduates and clinical pharmacists is appropriate knowledge and skills in pharmaceutical care. Clinical pharmacists directly participate in the treatment together with doctors, which is conducive to pharmacists’ more detailed understanding of patients’ conditions, training clinical thinking ability, and accumulation of innovation ability. At the same time, it can better understand the real needs of doctors and patients, sort out the standardized process, and realize the innovation of management system and model. Another hand, by carrying out joint outpatient clinics, clinical pharmacists and physicians can work together to find medication errors in time, put forward rational medication suggestions, reduce medication risks, and jointly improve service capabilities. Of course, the construction of pharmacy is inseparable from the support of hospitals and national policies.

Another important point mentioned in the interview is that if the status and responsibilities of pharmacist cannot be guaranteed through strong legal and regulatory documents, then the landing of relevant policies in medical institutions is also difficult to maintain. At present, the Ministry of Health have issued policies regarding the implementation of Pharmaceutical and Therapeutics Committees (PTCs) and the employment of clinical pharmacists to encourage appropriate prescribing (29, 30). Recently, the “China Clinical Pharmacist Core Competency Framework Expert Consensus (2023)” was released, which establishes the core competency framework that clinical pharmacists should possess (31). These policies have recognized the importance of clinical pharmacists in healthcare settings. However, the current performance appraisal of pharmacist cannot reflect the value of pharmaceutical care, which has seriously affected the internal motivation and work enthusiasm of pharmacist. The interviewees are eager to promote the “Chinese Pharmacist Law,” which is not only a separate law and regulation, but also linked to other policy documents related to rational drug use. When the policy document is issued, the responsibilities and obligations of the pharmaceutical department will be clearly defined. On the one hand, to enhance the social recognition of pharmacists, help the process of self-improvement of pharmacists, and on the other hand, to facilitate the implementation and supervision of relevant policies on rational drug use in the future.

However, this study also has several limitations. First, the initial interviews were transcribed and then translated from Chinese to English, which is a second language for all of the authors. Second, the study has limitations owing to qualitative research design. The participant number was small, leading to a potential risk of selection bias. Thirdly, the subjective assessments of participants may not accurately reflect the real situation. Some negative feelings may be underestimated or magnified. And the progress in each city implementing DRG/DIP was different, so the study results at this point in the time may be inconsistent with a full DRG/DIP implementation. However, we still believe that our results are representative and reliable, as we surveyed eight different nationwide cities in China, although further studies with larger sample sizes are needed in the future.

## 5 Conclusion

After the interview, we gained insights into the current work status of clinical pharmacists operating within the DRG payment system. The implementation of the DRG payment system has had a some impact on their work. However, there is still a need to enhance their understanding and active involvement in the policy. The greater involvement of clinical pharmacists in medical insurance work still requires policy support at the healthcare structure and national levels. Further standardization of pharmaceutical services and improvement in the motivation of practitioners can be achieved through reasonable performance assessments. At the same time, clinical pharmacists also need to enhance their own professional service capabilities. The results obtained after conducting a qualitative interview among clinical pharmacists can help identify the current situation of pharmaceutical care under DRG/DIP, possible problems during clinical practice, thereby contributing to further development of DRG in China.

## Data availability statement

The original contributions presented in the study are included in the article/supplementary material, further inquiries can be directed to the corresponding authors.

## Author contributions

SG: Investigation, Writing – original draft, Data curation, Methodology. XW: Investigation, Writing – review & editing. YLu: Methodology, Writing – review & editing. YLi: Investigation, Writing – review & editing. QJ: Methodology, Writing – review & editing. JF: Project administration, Writing – review & editing. WK: Project administration, Writing – review & editing. LL: Writing – review & editing. HC: Writing – review & editing, Project administration, Writing – original draft, Methodology.

## References

[1] Dieleman JL Templin T Sadat N Reidy P Chapin A Foreman K. National spending on health by source for 184 countries between 2013 and 2040. Lancet. (2016) 387:2521–35. doi: 10.1016/S0140-6736(16)30167-2, PMID: 27086174

[2] Jakovljevic M Zugic A Rankovic A Dagovic A. Radiation therapy remains the key cost driver of oncology inpatient treatment. J Med Econ. (2015) 18:29–36. doi: 10.3111/13696998.2014.971162, PMID: 25268728

[3] Administration NHS. Notification of national pilot cities for DRG payment. (2020) Available at: http: //www.nhsa.gov. cn/art/2019/6/5/art_37_1362.html.

[4] Kwon S. Payment system reform for health care providers in Korea. Health Policy Plan. (2003) 18:84–92. doi: 10.1093/heapol/18.1.84, PMID: 12582111

[5] Chang WF Yan XY Ling H Liu T Luo AJ. A study of the types and manifestations of physicians' unintended behaviors in the DRG payment system. Front Public Health. (2023) 11:1141981. doi: 10.3389/fpubh.2023.1141981, PMID: 37441652 PMC10333571

[6] McCue MJ Thompson JM. Early effects of the prospective payment system on inpatient rehabilitation hospital performance. Arch Phys Med Rehabil. (2006) 87:198–202. doi: 10.1016/j.apmr.2005.10.029, PMID: 16442972

[7] Zou K Li HY Zhou D Liao ZJ. The effects of diagnosis-related groups payment on hospital healthcare in China: a systematic review. BMC Health Serv Res. (2020) 20:112. doi: 10.1186/s12913-020-4957-5, PMID: 32050962 PMC7017558

[8] Sandelowski M. Whatever happened to qualitative description? Res Nurs Health. (2000) 23:334–40. doi: 10.1002/1098-240x(200008)23:4, PMID: 10940958

[9] Eryang C Yuye H Pharmacy DOJCP. Survey on the cognition of medical staff to clinical Pharmacy work in primary hospitals. China Pharm. (2018) 21:1041–3.

[10] Feng Y Bing L Caijun Y Jie C Tao Z Zhen J,Pharmacy management and pharmaceutical service model in hospitals of China. (2016) 25:6–9.

[11] Perepelkin J Dobson RT. Influence of ownership type on role orientation, role affinity, and role conflict among community pharmacy managers and owners in Canada. Res Social Adm Pharm. (2010) 6:280–92. doi: 10.1016/j.sapharm.2009.11.001, PMID: 21111386

[12] Li W Lin G Xu A Huang Y Xi X. Role ambiguity and role conflict and their influence on responsibility of clinical pharmacists in China. Int J Clin Pharm. (2020) 42:879–86. doi: 10.1007/s11096-020-01053-w, PMID: 32405715

[13] Council on Credentialing in Pharmacy Albanese NP Rouse MJ. Scope of contemporary pharmacy practice: roles, responsibilities, and functions of pharmacists and pharmacy technicians. J Am Pharm Assoc. (2010) 50:e35–69. doi: 10.1331/JAPhA.2010.10510, PMID: 20199947

[14] Qing F Ce Z. Development and Prospect of Clinical Pharmacists in China. J China Pharmacy. (2008) 65–7.

[15] Sanborn MD. Population health management and the pharmacist's role. Am J Health Syst Pharm. (2017) 74:1400–1. doi: 10.2146/ajhp170157 28887340

[16] Watanabe JH. Pharmacist-directed care to optimize medication use: a healthcare imperative in the United States. Expert Rev Pharmacoecon Outcomes Res. (2020) 20:419–21. doi: 10.1080/14737167.2020.1820865, PMID: 32902351

[17] McKnight AG Thomason AR. Pharmacists' advancing roles in drug and disease management: a review of states' legislation. J Am Pharm Assoc. (2003). 2009) 49:554–8. doi: 10.1331/JAPhA.2009.08056, PMID: 19589769

[18] Auta A Maz J Strickland-Hodge B. Perceived facilitators to change in hospital pharmacy practice in England. Int J Clin Pharm. (2015) 37:1068–75. doi: 10.1007/s11096-015-0153-9, PMID: 26195124

[19] Furuhata H Araki K Ogawa T Ikeda M. Effect on completion of clinical pathway for improving clinical indicator: cases of hospital stay, mortality rate, and comprehensive-volume ratio. J Med Syst. (2017) 41:206. doi: 10.1007/s10916-017-0857-6, PMID: 29134334

[20] Askari M Tam J Klundert J. The effectiveness of clinical pathway software in inpatient settings: a systematic review. Int J Med Inform. (2021) 147:104374. doi: 10.1016/j.ijmedinf.2020.104374, PMID: 33422761

[21] Park S Kim S Kim HB Youn SW Ahn S Kim K. Effects of implementing a clinical pathway on antibiotic prophylaxis for patients who underwent an elective surgery. Sci Rep. (2022) 12:20176. doi: 10.1038/s41598-022-24145-1, PMID: 36418406 PMC9684115

[22] Tan Z Yu Z Chen K Liu W Zhao R. Effects of pharmacist-led clinical pathway/order sets on cancer patients: a systematic review. Front Pharmacol. (2021) 12:617678. doi: 10.3389/fphar.2021.617678, PMID: 34093177 PMC8176097

[23] Wang R Liu B Feng X Tang B Chen B He Y. The effect of pharmacist-initiated perioperative multidisciplinary pharmaceutical care model and clinical pathway on pain management in patients undergoing orthopedic surgery: a before-after study. Int J Clin Pharm. (2023) 45:929–39. doi: 10.1007/s11096-023-01575-z, PMID: 37165280

[24] Liao X Guo WD Cao Z Xu HY Zhang YL Zhao H. Study on establishing a methodology of comprehensive clinical evaluation for Chinese patent medicine based on health technology assessment. Zhongguo Zhong yao za zhi = Zhongguo zhongyao zazhi = China journal of Chinese materia medica. (2020) 45:3749–58. doi: 10.19540/j.cnki.cjcmm.20200624.501 32893567

[25] Friedmann C Levy P Hensel P Hiligsmann M. Using multi-criteria decision analysis to appraise orphan drugs: a systematic review. Expert Rev Pharmacoecon Outcomes Res. (2018) 18:135–46. doi: 10.1080/14737167.2018.1414603, PMID: 29210308

[26] Yu Y Jia L Meng Y Hu L Liu Y Nie X. Method development for clinical comprehensive evaluation of Pediatric drugs based on multi-criteria decision analysis: application to inhaled corticosteroids for children with asthma. Paediatr Drugs. (2018) 20:195–204. doi: 10.1007/s40272-017-0278-5, PMID: 29247424

[27] Fang Y Yang S Zhou S Jiang M Liu J. Community pharmacy practice in China: past, present and future. Int J Clin Pharm. (2013) 35:520–8. doi: 10.1007/s11096-013-9789-5, PMID: 23661172

[28] Jiang JH Liu Y Wang YJ Liu X Yang M Zeng Y. Clinical pharmacy education in China. Am J Pharm Educ. (2011) 75:57c. doi: 10.5688/ajpe75357c, PMID: 21655413 PMC3109812

[29] China MoHotPsRo. Hospital reform implementation guidelines from the Ministry of Health. (2011). Available at: http://www.moh.gov.cn/yzygj/s3585u/201104/c6fa4cc981d4429ba8caa7666aa13710.shtml.

[30] China MoHotPsRo. Policy on pharmacy administration in health care facilities. (2011). Available at: http://www.nhc.gov.cn/yzygj/s3585u/201104/c6fa4cc981d4429ba8caa7666aa13710.

[31] Peking union medical college Hospita CAoMSPUMC, China Medical Board, Chinese hospital association pharmaceutical specialized committee. Expert consensus on the Core competency framework of Chinese clinical pharmacist. Medical Journal of Peking Union Medical College Hospital. (2023) 14:257–65. doi: 10.12290/xhyxzz.2023-0092

